# The Plant Viruses and Molecular Farming: How Beneficial They Might Be for Human and Animal Health?

**DOI:** 10.3390/ijms24021533

**Published:** 2023-01-12

**Authors:** Gergana Zahmanova, Alaa A. Aljabali, Katerina Takova, Valentina Toneva, Murtaza M. Tambuwala, Anton P. Andonov, Georgi L. Lukov, Ivan Minkov

**Affiliations:** 1 Department of Plant Physiology and Molecular Biology, University of Plovdiv, 4000 Plovdiv, Bulgaria; 2 Department of Technology Transfer and IP Management, Center of Plant Systems Biology and Biotechnology, 4000 Plovdiv, Bulgaria; 3 Department of Pharmaceutics and Pharmaceutical Technology, Faculty of Pharmacy, Yarmouk University, Irbid 21163, Jordan; 4 Institute of Molecular Biology and Biotechnologies, 4108 Markovo, Bulgaria; 5 Lincoln Medical School, University of Lincoln, Brayford Pool Campus, Lincoln LN6 7TS, UK; 6 Department of Medical Microbiology and Infectious Diseases, Max Rady College of Medicine, University of Manitoba, Winnipeg, MB R3T 2M9, Canada; 7 Faculty of Sciences, Brigham Young University–Hawaii, Laie, HI 96762, USA

**Keywords:** plant molecular farming, virus-like particles (VLPs), protein cages, plant-derived vaccines, biologics, nanomedicine, viral-based nanotechnology, drug delivery, immune therapy

## Abstract

Plant viruses have traditionally been studied as pathogens in the context of understanding the molecular and cellular mechanisms of a particular disease affecting crops. In recent years, viruses have emerged as a new alternative for producing biological nanomaterials and chimeric vaccines. Plant viruses were also used to generate highly efficient expression vectors, revolutionizing plant molecular farming (PMF). Several biological products, including recombinant vaccines, monoclonal antibodies, diagnostic reagents, and other pharmaceutical products produced in plants, have passed their clinical trials and are in their market implementation stage. PMF offers opportunities for fast, adaptive, and low-cost technology to meet ever-growing and critical global health needs. In this review, we summarized the advancements in the virus-like particles-based (VLPs-based) nanotechnologies and the role they played in the production of advanced vaccines, drugs, diagnostic bio-nanomaterials, and other bioactive cargos. We also highlighted various applications and advantages plant-produced vaccines have and their relevance for treating human and animal illnesses. Furthermore, we summarized the plant-based biologics that have passed through clinical trials, the unique challenges they faced, and the challenges they will face to qualify, become available, and succeed on the market.

## 1. Introduction

Since plant viruses were discovered in the late nineteenth century, their impact has changed from harmful pathogens to useful molecular machines with applications in plant molecular farming (PMF) and bio-nanotechnology. Although public perception of a “virus” is often associated with harm, viruses can easily be reorganized into environmentally friendly and safe structures [1,2,3]. The expansion of the overall knowledge and understanding of viral genomes, architecture, and biophysical properties has allowed the use of plant viruses as vectors for recombinant protein expression and the production of virus-based nanoparticles (VNPs) [4]. VLPs are self-assembled, naturally occurring nanomaterials that structurally and morphologically resemble the 3-dimensional structures of virions, but without the viral genome. VLPs modified to carry additional useful payload are defined as VNPs. Some of the many biological applications of VNPs, which are generated from the capsid structural components of various viruses, include vaccine production and innovative delivery mechanisms for therapeutic compounds, diagnostic reagents, enzymes, pesticides, immunogenic peptides, and many others [5].

In the present review, we will cover the latest developments in PMF and the usefulness of plant viruses in the production of bio-nanomaterials for human and animal health. We will also discuss virus-like nanoparticle-based technologies for vaccine production in plants and the possibility of using unpurified materials for oral treatment. Furthermore, we highlight the usefulness of plant-derived VNPs as templates, carriers, containers, and scaffolds for drug delivery and application in nanomedicine.

### Historical Review of Plant Molecular Farming

**Figure 1:**
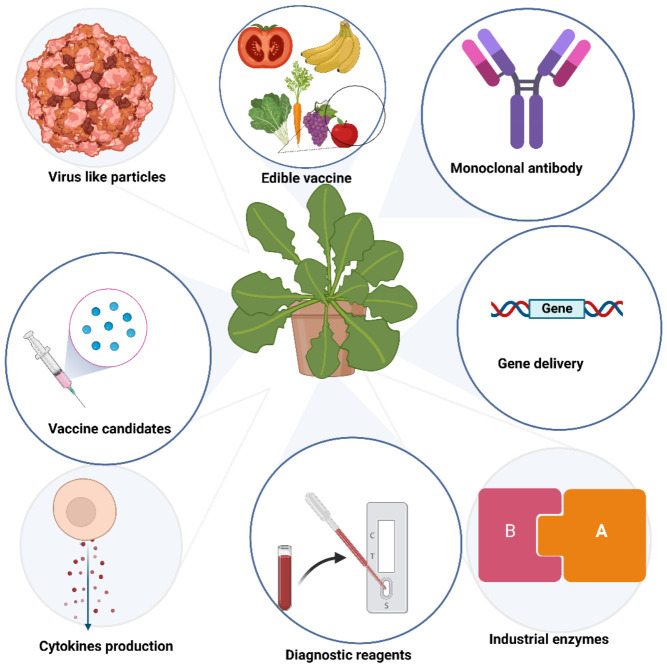
Biologics produced in plants.

Over the past three decades, several plant systems have been used to produce recombinant proteins. Much interest has been paid to plant-derived antibodies, vaccines, enzymes, microbicides, and viral protein nanocages (Figure 1).

Plant molecular farming was born in 1980 when the marker gene for β-glucuronidase (GUS) was successfully transformed in higher plants [6]. Later, β-glucuronidase became one of the successful commercial products of plant molecular farming, together with two other industrial enzymes, avidin and trypsin [7,8,9]. In 1986, human growth hormone (HGH) was produced in transgenic tobacco and sunflowers [10]. In 1988, the first production of human antibodies in plants was reported [11]. The concept of using plants for the production of vaccines, more specifically, edible vaccines, was introduced by Dr. Charles Arntzen in the 1990s [12]. Studies were focused on this topic for many years [13,14,15] until the incident of seed contamination with a transgene expressing a bovine vaccine candidate [16,17]. The new trends in plant molecular farming are to use plants in controlled conditions to produce recombinant products that can either be injected or used orally [18,19,20,21,22].

The initial plant-derived biopharmaceuticals were expressed in stable transgenic plants. This technological approach has three main disadvantages: a long developmental period, low yield, and public concerns about GMO plants. Transient expressions via plant expression virus vectors offer a way to avoid these limitations [23]. 

Although the production of recombinant proteins in plants by stable gene integration and expression has some limitations, the initial commercialized therapeutic recombinant proteins were produced by this technology. In 2006, stably transformed *N. benthamiana* cell cultures were used for the production of the first USDA-approved injectable vaccine against the Newcastle disease virus for poultry [24]. In 2012, the FDA approved plant-derived recombinant human β-glucocerebrosidase (marketed as Elelyso^®^) for the treatment of Gaucher’s disease type 1. The recombinant human β-glucocerebrosidase enzyme was produced in a stably transformed carrot cell suspension culture by Protalix Biotherapeutics Inc. [25] and licensed to Pfizer. In 2022, Health Canada approved the first plant-derived SARS-CoV-2 vaccine, Covifenz [26]. The strategy included transient expression in *N. benthamiana* of the modified recombinant SARS-CoV-2 S protein with stabilizing point mutations. Medicago Inc. successfully produced and purified modified SARS-CoV-2 S VLPs built from the S protein. Using the same technology, Medicago Inc. successfully produced a vaccine against seasonal influenza [27]. Currently, there are few examples of commercialized plant molecular farming products, and plants cannot replace industrial expression systems. However, PMF has been proven cost-effective and valuable in the production of vaccines, antibodies, enzymes, and biological VNPs under conditions of urgency, such as emerging pandemic viruses or for treating rare diseases.

## 2. Important Plant Viruses for Plant Biotechnology 

**Figure 2:**
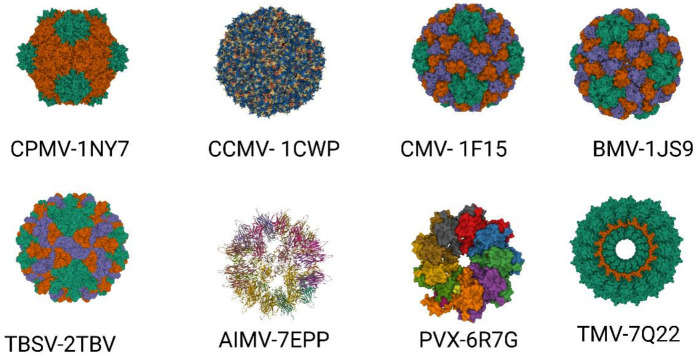
Important plant VLPs with application in biotechnology and plant viral-based nanotechnology. Plant viruses with their corresponding protein databank identification number—CPMV (PDB ID: 1NY7), CCMV (PDB ID: 1CW7), CMV (PDB ID: 1F15), BMV (PDB ID: 1JS9), TBSV (PDB ID: 2TBV), AIMV (PDB ID: 7EPP), PVX (PDB ID: 6R7G), TMV (PDB ID: 7Q22).

Approximately 900 plant viruses, identified mainly in crops, are known [28]. Several of them, such as the tobacco mosaic virus (TMV), potato virus X (PVX), cowpea mosaic virus (CPMV), cowpea chlorotic mottle virus (CCMV), brome mosaic virus (BMV), cucumber mosaic virus (CMV), plum pox virus (PPV), alfalfa mosaic virus (AIMV), papaya mosaic virus (PapMV), tomato bushy stunt virus (TBSV), and many others, are utilized in biotechnology and plant viral-based nanotechnology research and industry (Figure 2) [29,30].

Plant viruses have very simple (rod-shaped, spherical, quasi-spherical, or filamentous) structures, consisting of multiple copies of one or a few capsid protein subunits, forming a protein coat around the viral genome. The capsid proteins have the ability to self-assemble, which offers the opportunity to generate a great variety of natural bio-nanomaterials. A better understanding of the structure and biophysical characteristics of plant viruses is vital for developing and producing VNPs, which can be used as nanoparticles for the surface presentation of antigens for recombinant VNP vaccines or as nanocages for “cargo” delivery. VNPs are naturally occurring biological entities, making them both biocompatible and biodegradable. Furthermore, VNPs can be targeted to particular tissue or cells. Finally, the production and purification of VNPs from plants are rapid, cost-effective, and environmentally safe (compared with conventional nanoparticles) and can easily be scaled up depending on demand [31]. There are two main strategies for VNPs production in plants: using a virus infection, allowing the cultivation of plant viruses in their natural host, or the biotechnology approach, using transient expression of the viral genes encoding the capsid proteins in heterologous expression systems [32,33,34].

### 2.1. Tobacco Mosaic Virus (TMV)

TMV is a member of the genus *Tobamovirus* in the family of *Virgaviridae***.** TMV has a rod-shaped virion with a diameter of 18 nm, a modal length of 300 nm with a central channel in width, and the viral RNA intercalated between the coat protein turns [35]. TMV coat protein (CP) could self-assemble into the form of a rigid helical RNA-free tube [36]. Tobacco mosaic virus can be easily cultivated in plants, and the yields can be very high, ~4 g/kg wet weight of tobacco. The purified TMV rod-shaped virions can be disassembled in vitro into protein subunits and RNA [37,38]. When nucleic acid is absent, the coat protein can self-assemble into several types of VNPs. The polymerization of the TMV coat protein is temperature and concentration-dependent [39,40]. TMV VNPs are stable and can be used as scaffolds for chemical modifications, coating with biologically active peptides, or loading with drugs [17,41,42,43,44,45,46]. These modifications will be discussed in greater detail in Section 6 of this review. TMV has been widely used as a full-vector and also as a deconstructed virus vector for recombinant protein expression [47].

### 2.2. Cowpea Mosaic Virus (CPMV)

CPMV belongs to the genus *Comovirus* in the *Comoviridae* family. It is a non-enveloped, icosahedral virus with nanoscale dimensions (30 nm). The CPMV protein shell is comprised of 60 copies of the large (L) coat proteins with two domains and 60 copies of the small (S) coat proteins with one domain [48]. The three domains together form the asymmetric unit of the CPMV capsid, with 2 nm channels enabling the exchange of molecules from the exterior to the interior. The internal viral cavity encapsulates two single-stranded RNA molecules [49,50].

This CPMV virus has been widely used in bio-nanotechnology because its structure and particle architecture have been well studied, and the genome can easily be manipulated [51]. Virus particles, which contain viral genomic RNAs, can be produced rapidly in high yield (1–2 g/kg) through the infection of plants [48]. These viral particles have been used for selective attachment of various moieties [51,52,53,54,55] and to display immunogenic epitopes on their surface [56,57]. The drawback is that the CPMV particles containing genomic RNAs raise biosafety and regulatory concerns. They are also ineffective drug containers because they are hard to load with foreign materials such as therapeutic agents or heterologous RNA molecules. Prof. Lomonossoff’s laboratory addressed these problems by developing RNA-free, empty virus-like particles (eVLPs) based on the transient expression of VP60 (precursor of L and S coat proteins) along with the 24K viral proteinase in *Nicotiana benthamiana* [58,59,60,61]. In addition, the eVLPs are very stable under various conditions, which extends their bio-nanotechnology application range [2,61,62,63]. Lomonossoff’s lab further improved CPMV viral particle packaging, allowing the production of VLPs that can pack up to 6 kb of artificial RNA [64]. Furthermore, CPMV was used for the expression of recombinant proteins by applying full-virus and deconstructed virus strategies [65].

### 2.3. Cowpea Chlorotic Mottle Virus (CCMV)

CCMV belongs to the *Bromoviridae* family. The CCMV capsid is composed of 180 identical capsid proteins (CP) that form a ~28 nm diameter icosahedral shell and an 18 nm diameter inner cavity [66]. Native CCMV is stable at pH 5.0. The native CCMV virion can be disassembled, and the RNA genome can be removed by centrifugation under high salt concentrations at neutral pH [67]. In vitro, at pH 5.0, the CCMV capsid proteins can be self-assembled into empty nanoscale structures [68]. CCMV is one of the first viruses used in bio-nanotechnology as a tool for developing drug-delivery vehicles due to its well-studied structure, simple capsid with good biocompatibility, and low toxicity [69]. VNPs based on CCMV have been used to encapsulate guest molecules, such as negatively charged polymers, enzymes, and organic aggregates [70]. A wide variety of ligands, such as small peptides, biotin, fluorescent dyes, organometallic photosensitizers, and intact IgG antibodies, can be attached on the outer surface of the capsid [71,72,73,74].

### 2.4. Brome Mosaic Virus (BMV)

BMV, a member of the family *Bromoviridae*, is very similar to CCMV [75]. The native virion has a diameter of 28 nm with T = 3 icosahedral symmetry, and it is built of 180 identical capsid proteins with different conformations (A, B, and C) [76,77]. The A conformation of the coat protein forms pentameric capsomers with small 0.5 nm pores. The B and C conformations of the capsid protein form hexameric capsomer with 0.6 nm pores [76]. The stability of the capsid depends on the interaction between the positively charged inner cavity and the negatively charged encapsulated RNA [78]. BMV, similarly to CCMV, is widely used in bio-nanotechnology due to its well-examined structure and biophysical properties.

## 3. Plant Viruses as a Source of Vectors for Transient Expression

The use of plant viruses as vectors for delivering foreign genes into plants began in the 1980s [79,80]. Understanding the viral genomes’ structure and the strategies plant viruses use for their gene expression is essential for constructing and improving virus-based expression systems. The plant virus-based expression systems possess many advantages, including rapid and high-level gene expression and a reduced risk for contamination with transgenes. Cauliflower mosaic virus (CaMV) was used to construct the first plant virus expression systems. The limitation of these expression systems was their small capacity to integrate foreign genes after removing large regions of their viral DNA [81].

A major breakthrough in the development of plant virus-based expression systems was the use of single-stranded RNA viruses and *Agrobacterium tumefaciens* to insert viral genes into the plant cell [82]. The potential of many plant viruses to become a source of useful vectors for plant genome engineering has been investigated (Table 1). The most well-known of these are TMV [83], PVX [84], CPMV [85], tobacco rattle virus (TRV) [86], barley stripe mosaic virus (BSMV) [87], and many others.

The plant virus-based vectors can be divided into first-generation (gene substitution vectors, gene insertion vectors, and gene display vectors) and second-generation (deconstructed vector systems) expression vectors. 

First-generation expression vectors use the entire viral genome. The gene of interest is expressed as a substitute coat protein, fused to a coat protein, or separated into another open reading frame (ORF) under the control of a potent subgenomic promoter [97]. The use of gene replacement vectors is limited because of their inability to induce systemic infections and cell-to-cell movement due to the substitution of endogenous viral sequences of coat proteins with heterologous genes of interest [47]. Gene insertion vectors contain complete functional viruses with additional ORFs for recombinant proteins. These vectors can support cell-to-cell movement and induce systemic infection. TMV and PVX are the two viruses most commonly used to create gene insertion vectors [98,99]. Gene fusion vectors are constructed by fusing a recombinant peptide of interest with a coat protein. The early works with genetically modified coat proteins of several plant viruses supporting heterologous peptide fusions and exposing them on the virion surface have been reviewed [100,101].

With the introduction of second-generation virus vectors, transient expression effectiveness has improved drastically. These vectors contain deconstructed viral genomes, keeping only essential and some additional components needed for effective protein expression [83,102]. The constructed modular system consists of the main viral components separated into distinct parts and inserted into binary vectors within *Agrobacterium tumefacience*. By shortening the vector size, the length of the desired gene is extended. Since the genes essential for virus transport and assembly have been removed, deconstructed virus vectors are delivered to plants primarily via vacuum agroinfiltration, leading to simultaneous expression of the protein of interest in all plant tissues with an optimal harvest yield [103]. In addition, the co-expression of vectors containing different structural parts can result in the construction of macromolecular protein structures such as empty virus-like particles (eVLPs) [59].

At present, numerous recombinant proteins used as human and veterinary pharmaceuticals have been produced using plant-based transient expression systems such as magnICON^®^ [104], potato virus X-based vectors [105,106], and the cowpea mosaic virus-based vector pEAQ-*HT* [107,108,109,110,111]. These vectors made it possible for recombinant protein expression levels to reach more than 40% of total soluble protein [112]. 

## 4. Functionalization Methods of VNPs

**Figure 3:**
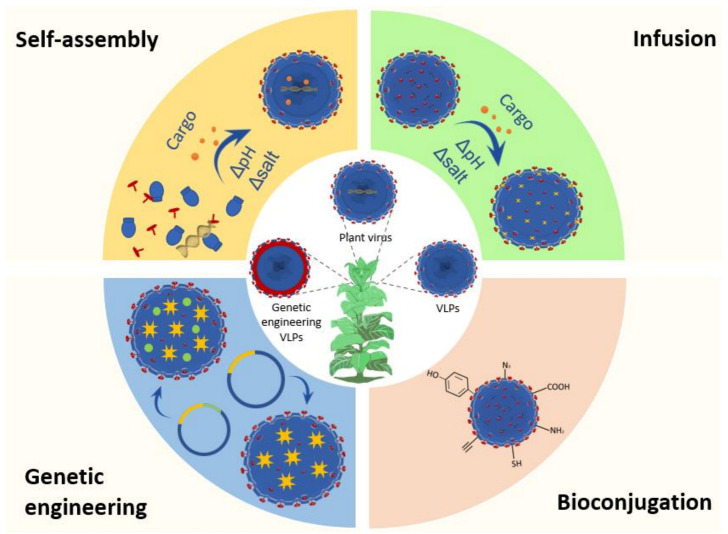
Strategies for functionalization of VNPs.

Plant viruses are intriguing because their coat proteins may construct robust, biodegradable delivery systems for a wide range of chemicals by self-assembling around natural and synthetic molecules of interest. Viruses employ various strategies to self-assemble coat proteins around cargo moieties. For several viral platforms, encapsulating foreign materials is dependent on the interaction of coat proteins with a particular sequence of the viral RNA (i.e., red clover necrotic mosaic virus (RCNMV)) or a negatively charged substance (i.e., BMV, CCMV, Hibiscus chlorotic virus (HCRSV)) to replace the negatively charged RNA [113]. Disassembly and reassembly strategies can be used to release the viral genome and encapsulate new cargo (Figure 3).

The viral genome can be released from the capsid by altering the in vitro buffer conditions, pH, and ionic strength [114,115]. Using buffer exchange techniques, viral capsids are disassembled and then reassembled to enclose the target payload. To give just one example, CCMV disassembles and releases its core RNA at physiological pH or high ionic strength (1M) but reassembles in a buffer with a pH between 3 and 6 and low ionic strength (0.1 M) [116]. Like the creation of micelles, reassembly can be induced by combining the cargo with coat proteins at a concentration higher than the critical assembly concentration of the VLPs. Cargo encapsulation success is influenced by a variety of parameters, including, but not limited to, cargo size and surface charge, electrostatic interactions, hydrophobicity, and hydrophilicity [117].

However, with the simple co-expression of necessary viral proteins in plant cells, certain virus particles (CPMV) may form hollow capsids in the absence of genetic/external material. Molecules that are small enough and can pass through the pores of the viral capsid can also be encapsulated [118]. However, this mechanism depends on the size of the molecules and the diameter of the pores, meaning that various viruses will have different packing abilities. For some viruses, pore development can be stimulated by removing divalent ions from inside the capsid [119,120,121,122]. This feature of RCNMV was illustrated by Lommel et al. [123] by removing calcium and magnesium ions from the solvent using chelators, which resulted in the development of channels with a diameter of 11–13 Å. Re-addition of the ions closed the created channels.

By manipulating the buffer conditions, CCMV may be produced in an open and expanded configuration, creating holes of 2 nm that are suitable for cargo loading. Using a process called “gating,” the cargo is secured within by switching back to a closed and condensed shape [124]. This diffusion method has also been used with the artichoke mottled crinkle virus (AMCV) [125] and Johnson grass chlorotic stripe mosaic virus (JCSMV) [126] (Figure 3).

Other VNP-based strategies utilize the expression of fusion proteins on the exterior surface of VNP particles [127]. These chimeric proteins have been generated by fusing the coding sequences of a coat protein and a protein of interest (immunogenic peptides, receptors, tags, linkers, and others). While these gene-altering approaches generate new surface proteins, they do not disturb self-assembling during the expression in heterologous systems. Gene-engineered coat proteins are suitable for the bio-conjugation method for functionalizing the capsid. Bio-conjugation is joining biomolecules to other molecules forming a new complex of hybrid material. Targets for bio-conjugation are surface groups of reactive amino acid side chains, most commonly that of lysine (Lys), cysteine (Cys), or tyrosine (Tyr) aa, or linked carbohydrates of capsid proteins [128]. Bio-conjugation was used to functionalize VNPs for applications in drug delivery, imaging, theranostic, and immunotherapy [2,30,129,130].

## 5. Application of Plant VNPs for Delivering Bioactive Cargos

### 5.1. Anticancer Drugs

While conventional cancer therapies such as surgery, radiotherapy, and chemotherapy have a good track record of effectiveness, they also have some significant drawbacks. Although effective, conventional chemotherapy often has devastating side effects on healthy cells. The therapeutic outcomes of chemotherapy would be greatly improved if the chemotherapeutic agents were delivered in a targeted manner inside the tumor tissues. Such specific delivery of chemotherapeutics into the tumor cells would lead to a maximum therapeutic site with minimum toxic side effects. Combining conventional chemotherapy with biological treatments can not only deliver drugs in a highly targeted manner but can also boost antitumor immunity [131].

VNPs and VLPs have been used effectively in the delivery of the anticancer medication doxorubicin (DOX). DOX has been effectively delivered using TMV- and PVX-derived VLPs and VNPs. VNPs have shown a lot of promise since the simple adsorption of DOX on their surface, which effectively suppressed tumor development and progression [2]. RCNMV was coupled to a CD46-targeting polypeptide and packed it with DOX via passive diffusion of DOX through the constructed channels during the capsid’s “open” configuration. In vitro, HeLa cells were highly sensitive to the cytotoxicity of RCNMV NPs that had been targeted and loaded with DOX [132]. Peptides with targeted therapeutic effects against different malignancies can be administered through TMV. Phenanthriplatinn, reported as PhenPt-TMV, is an anticancer medication loaded into a hollow TMV vehicle. PhenPt-TMV is an example of a stimuli-responsive system in which the acidic microenvironment of the tumor drives the drug release. This is consistent with the findings of Tian et al., who showed that a Transacting Activation Transduction peptide conjugated to the TMV surface improved both internalization and the virus’s propensity to evade degradation in the endo- and lysosomal systems [133].

Platinum-derived medicines are used in half of the chemotherapy regimens. Targeted delivery of platinum-based anticancer drugs through plant virus VNPs and the effectiveness of TMV in transporting the platinum-based therapeutics cisplatin and phenanthriplatin have been reported. Both charge-driven interactions and the synthesis of stable covalent adducts were used to deliver the medicines into the TMV VNP cavity. It has been demonstrated in in vitro systems employing HepG2 and MCF-7 cancer cell lines that a TMV-based drug delivery system enables superior, targeted cytotoxicity and greater ease of absorption by cancer cells [134]. Mitoxanthrone, a topoisomerase II inhibitor, is another anticancer medication that has been reported to be encapsulated by TMV. Although direct administration of mitoxanthrone can have devastating cardiac side effects, VNPs carrying mitoxanthrone have been shown to significantly reduce tumor development in mouse cancer models [135]. Another example of a VNP-based anticancer drug delivery approach is the TMV VNPs specific for non-Hodgkin’s lymphoma that have the anticancer agent valine-citrulline monomethyl auristatin E (vcMMAE) attached to their surface. The efficacy of this approach was demonstrated in vitro against the Karpas 299 non-Hodgkin’s lymphoma cell line [45]. PVX displaying TNF related apoptosis induces ligand (TRAIL) stimulated death receptor recruitment and activation in HCC-38 primary ductal carcinoma, BT-549 ductal carcinoma, and MDA-MB-231 breast cancer cell lines [136].

### 5.2. Imaging Agents

VNPs can be developed for smart tissue-specific targeting of imaging agents. VNPs are nanoparticles that possess multivalent functional side chains of exposed amino acids that can be used as anchoring points for imaging modalities. Imaging molecules can be attached to the outside surface or inside cavity of VNPs, through genetic engineering, by adding bioluminescent protein sequences to specific epitopes on the capsid, using bioconjugation chemistry, or infusion method.

Numerous in vivo imaging modalities are now in development. The most used in clinical practice are computed tomography (CT), magnetic resonance imaging (MRI), and positron emission tomography (PET). Magnetic resonance imaging and PET are used more frequently for cardiovascular applications and provide improved soft tissue imaging compared to CT. Both MRI and PET need the use of image-contrast agents—expensive isotopes such as 18F, 11C, or 15O in clinical applications and metals such as Gadolinium (GdIII) or iron for MRI—to achieve high sensitivity [137]. Using VNPs as frameworks for creating multivalent imaging sensors has several benefits: they can easily be functionalized through their surfaces’ exposed amino acid reactive groups to have better sensitivity and specificity, and possibly quicker response in medical analyses. 

The indocyanine green near-infrared imaging agent was encapsulated using VLPs based on BMV. The BMV capsid was doped with indocyanine green and used to image human bronchial epithelial cells with preserved cellular bioavailability over 3 h of incubation [138].

The fusion of the green fluorescent protein (GFP) or the red fluorescent protein, mCherry, with the N-termini of the PVX viral coat protein demonstrated that PVX can be used as an optical imaging agent in human cancer cells and mouse model studies [139,140]. TMV nanoparticles were modified to carry the oligopeptides (VHPKQHRAEEA-Lys(PEG7-N3)-NH2), which specifically recognized vascular cell adhesion molecule (VCAM)-1 on endothelial cells at atherosclerotic plaques. These modified TMV VLPs were loaded with near-infrared dyes and (Gd(DOTA)) for MIR. Using submicromolar dosages (400 times lower than the average therapeutic dose), the modified VLPs successfully recognized atherosclerotic plaques [141].

Labeling CPMV nanoparticles with the fluorescence fluorophore, AlexaFluor 555, at a ratio of 120 dyes per particle allowed scientists to image the vascular system in living animals. The resulting modified particle is brighter than orange-fluorescent microspheres of the same dimension and much brighter than fluorescent dextran. The entire circulatory system of a developing mouse was illuminated after the injection of labeled CPMV nanoparticles into the capillaries of the yolk sac. The injection of CPMV nanoparticles into a living chick embryo visualized the whole vascular system of the animal and allowed the observation of its vascular development over several days [142]. Fluorescently labeled CPMV particles, injected into a chick’s chorioallantoic membrane, visualized the developing vasculature of a tumor for at least 2 days. In addition, sequential inoculations with various fluorophore-enabled CPMV can visualize the developmental stages of a newly formed vasculature [142].

T2 imaging has also documented the development of viral scaffolds. The low signal strength of T2 shortening agents, in contrast to the vivid signal produced by T1 shortening agents, is a hallmark of these compounds. Nanoparticles consisting of iron oxide have been incorporated with VNPs as contrast agents. To accomplish imaging in the biomedical field, iron oxide NPs were encapsulated inside VLPs generated from BMV [143].

### 5.3. Nucleic Acid 

Nucleic acid-based therapeutics are a promising technology for treating several diseases and turned out to be the fastest and most effective way to create vaccines against emerging diseases. There are several categories of nucleic acid therapeutics: DNA/RNA coding functional proteins could overcome loss-of-function mutations; messenger RNA (mRNA) could induce translation and production of proteins; small interfering RNAs (siRNAs) and antisense oligonucleotides could induce gene silencing; and microRNAs (miRNAs) could modulate gene expression patterns [144,145]. siRNAs and miRNAs have a tremendous impact on cancer therapy and gene regulation. siRNAs became the first FDA-approved nucleic acid drugs [146]. Several delivery platforms have been used for nuclei acid therapeutics. They can be characterized into two groups: viral and non-viral nucleic acid delivery systems. Common vectors utilized for gene therapy are based on mammalian viruses, such as adenoviruses, adeno-associated viruses, lentiviruses, and retroviruses [147]. Non-viral delivery systems (liposomes, dendrimers, lipid nanoparticles (LNPs), and polymeric nanoparticles) are widely used due to their safety, easy modification, cell internalization, and allowing controlled releasing of the encapsulated therapeutics [148,149]. Viral and non-viral delivery systems have some limitations. Non-viral systems generally are more unstable, less accurate, and less effective than viral systems. The disadvantages of the viral vectors include the possibility of gene integration and pre-existing or innate immunogenicity [150,151,152]. Plant virus-based VNPs are easily reprogrammable nanoparticles with desired biological, chemical, and physical properties for effective nucleic acid delivery [153,154]. Using the disassembling/reassembling method, Steinmetz lab demonstrated that CCMV can encapsulate CpG oligodeoxynucleotides (ODNs). The direct injection of such particles into a tumor induced a robust antitumor response by increasing the phagocytic activity of macrophages [155]. 

Recently, CPMV coat protein was used to encapsulate and stabilize RNA molecules that can act as positive qRT-PCR controls for diagnosing viral diseases, including COVID-19 [156]. Such RNAs could either be used to induce gene silencing or act as mRNA to transiently produce proteins within the transfected cells. 

CCMV VNPs were loaded with heterologous RNA from mammalian Sindbis virus for delivering nucleic acids into mammalian cells [157]. Others proved that CCMV-based VNPs can transfect and deliver heterologous genes for translation into mammalian cells [158]. The CCMV coat protein was used to encapsulate and deliver siRNAs into mammalian cells for gene knockdown [159]. Furthermore, CCMV was used as a platform for delivery of self-amplifying mRNA vaccine [160].

TMV was used to create a probe containing scrambled EBOV RNA sequences that can be used as a positive control in Ebola RT-PCR diagnostics assay [161]. TMV was also used for direct gene delivery and expression into HeLa cells with proven expression of TMV coat protein [162].

### 5.4. Non-Biological Synthetic Nanoparticles

Material science and medicine can greatly benefit from chemically synthesized NPs (metallic, magnetic, and semiconducting polymers). The ideal NPs for human and animal use should be safe, biocompatible, biodegradable, and tailored to the area in need of improvement. Covering these man-made NPs with proteins found on the outside of plant virus coats can make them more biocompatible, improve their delivery, and get rid of them faster.

**Figure 4:**
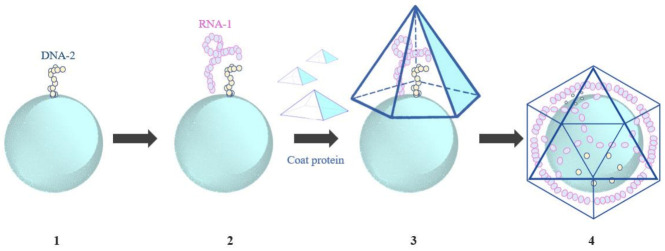
Mechanism of reassembly of RCNMV coat protein around metal nanoparticles [163]. (**1**) metal nanoparticles were linked with synthetic oligonucleotides (DNA-2) mimicking the OAS, (**2**) RCNMV RNA-1 was used to interact with DNA-2, (**3**) OAS formation, (**4**) reassembly of RCNMV coat proteins [163].

The red clover necrotic mosaic virus (RCNMV) has been reported to encapsulate tiny gold, cobalt ferric oxide, and cadmium selenide nanoparticles (varying in size from 3 to 15 nm) [163]. The oligonucleotides mimicking the DNA framework and origin of assembly (OAS) were used to join the metal nanoparticles. The assembly of VLPs is initiated by OAS when interacting with viral coat proteins. Loo et al. introduced RCNMV RNA-1 using synthetic oligonucleotides. This led to the formation of OAS and the assembly of RCNMV CPs around metal nanoparticles, leading to the formation of VNPs, as shown in Figure 4. 

Using polyethylene glycol (PEG)ylated phospholipids containing terminal carboxyl groups, Huang et al. documented how iron oxide NPs of about 20.1 nm in size can create VNPs. The capsid proteins of the Brome mosaic virus (BMV) were able to wrap metal NPs in a way that made the VNPs’ size 41 nm [143]. This research also revealed that BMV capsids can be used to incorporate foreign material into plant cells. Using VNPs as MRI probes to track vital plant functions is another area pioneered by Huang’s team [143].

Prussian blue (PB) was encapsulated by CCMV capsid proteins due to electrostatic repulsion. PB, a metal complex with a negative charge, was encapsulated via a disassembly/reassembly approach in which the pH was dropped from 7.5 to 5.2. Bimetallic nanoparticles were efficiently self-organized because of PB’s encapsulation and crystallization in CCMV [164]. 

The CCMV coat protein was expressed in the yeast *Pichia pastoris* and purified before being reassembled into CCMV empty capsids. The CCMV virus itself contains a metal-binding domain that has been found to bind to Terbium (III). It was shown that the particles could bind Gd^3+^, creating paramagnetic nanoparticles with relaxivity measurements 5–10 times greater than any of those recorded for Gd^3+^-albumin or Gd^3+^-dendrimers [165,166].

Unique nanomaterials and nanotechnologies can be created by depositing materials, including Ag, Ni, Co, Cu, Pt, and Fe-Pt alloy, into the 4 nm core cavity of TMV [167,168,169]. Even more so, Kobayashi et al. employed genetically engineered tobamoviruses to generate magnetized 3 nm tube-shaped VNPs. Tomato mosaic virus (ToMV) central channel amino acid residues were genetically modified to increase the amount of positive charge nucleation sites to absorb precursor cations. This led to the incorporation of a CoPt alloy into the inner channels within the ToMV [170]. Tobamovirus VNPs are an exciting new way to make a wide variety of nanoscale building blocks that can be used to make electronics.

The templated manufacturing of highly dispersed gold nanoparticles describes an innovative use of polyelectrolyte-modified Cowpea mosaic virus (CPMV). Poly(allylamine) hydrochloride (PAH), a cationic polyelectrolyte, is electrostatically attached to the exterior of the virus capsid, where it facilitates the adsorption of anionic gold complexes that can be readily reduced under mild conditions to generate a metallic gold coating. Thiol reagents allow for further modification of the templated gold nanoparticles. When polyelectrolyte-modified CPMV (CPMV-PA) reacts with already-formed gold nanoparticles, large, hexagonally-packed, tessellated spheres form on their own [171].

Their distinctive material properties and larger surface area may provide higher activity over comparable bulk materials, making them useful in a variety of industrial applications [172]. Altering the catalyst’s positioning, size, and spatial density is also a powerful tool for managing the reactions that take place during catalysis. PVNs have served as a novel scaffold for the fabrication, anchoring, and exposition of essential nanocatalysts. TMV PVN was used by Yang et al. to generate and distribute Pd nanoparticles with a predetermined size (between 5 and 15 nm) [173]. When used to break down hexavalent chromium (environmental pollutant), the TMV-patterned Pd nanocatalysts had much higher catalytic activity, per unit of Pd bulk, than commercial Pd carbon systems.

The application of plant viruses in the production of nanomaterials and nanodevices has been reviewed in several papers [124,172,174].

### 5.5. Plant-Derived VNPs Loading Enzymes

Conjugation of enzymes with plant-derived VNPs is a highly beneficial approach for biosensing as it provides good accessibility of the active centers for the analyte molecules while ensuring temperature, pH, and proteases protection [174]. 

For example, TMV proved to be a suitable enzyme nanocarrier when biotin-streptavidin [SA] bioaffinity binding was applied to load glucose oxidase for amperometric detection of glucose [175]. The same principle was used to install a two-enzyme system, of glucose oxidase (GOx) and horseradish peroxidase (HRP), for the colorimetric detection of glucose. TMV tubes improved catalytic activities up to 45-fold compared to control samples with the same input of free enzymes [176]. Additionally, TMV nanorods loaded with [SA]-penicillinase (Pen) were successfully used for antibiotic detection because the integration increased the reusability and stability of the enzyme [177].

Potyvirus turnip mosaic virus (TuMV) also has great potential as a foreign epitope carrier: the virion’s external surface is rich in functional groups susceptible to chemical conjugations. In particular, Candida antarctica lipase B (CALB) conjugated onto the external surface of the TuMV demonstrated increased specific activity compared to the non-immobilized enzyme [178].

In addition, loaded VNPs with catalytic utilities can be used to activate drugs. For example, CCMV carrying CYP_BM3_ (a cytochrome P450 variant) is capable of transforming and activating the chemotherapeutic prodrug tamoxifen, the prodrug resveratrol, or similar products [179].

### 5.6. Plant-Derived VNPs Decorated with Antibodies and Nanobodies

Therapeutic and diagnostic applications of monoclonal antibodies (mAbs) are growing worldwide. The mAbs market is the largest sector of the global biopharmaceutical market, with sales exceeding over USD 100 billion worldwide [180], and is projected to reach USD 425 billion by 2028 [181]. Plants are an alternative source of recombinant mAbs [182]. Transgenic plants can provide large-scale production of recombinant mAbs, which are used for passive immunotherapy [182,183,184]. In addition, because of their high specificity and affinity binding, antibodies can be used to recognize and specifically bind to other molecules and, as such, become building blocks for nanomaterials. Antibodies can recognize a wide variety of targets, not only proteins and peptides but also small molecules (haptens), carbohydrates, and nucleic acids [185]. Smolenska et al. generated PVX particles linked with a scFv against the herbicide diuron [186]. The “overcoat strategy” was used to decorate the tobamovirus particles with the small protein A (33 amino acids). These modified nanoparticles were exploited for affinity purification of mAbs with a recovery yield of 50% and >90% purity [187]. Antibodies can not only be used for building simple or complex nanomaterials, but they can also add specific functional components to these materials. For example, with the help of antibodies, nanomaterials can gain the ability to bind and deliver specific structures to target cells, activate and recruit effector cells, or present specific enzymatic or sensor activities.

A new alternative to mAbs are the nanobodies, which are heavy-chain only antibodies (HcAbs) with a single variable domain (VHH, ~15kDa), produced in camels [188]. Nanobodies have great potential in therapeutics because they bind their epitopes with high specificity and strong affinity [189]. Plant-derived genetically modified VNPs from zucchini yellow mosaic virus (ZYMV) and tobacco etch virus (TEV) were used as scaffolds for nanobodies against the green fluorescent protein. The recombinant VNPs decorated with anti-GFP nanobodies were able to bind the GFP, demonstrating the efficacy of this technology [190]. This research demonstrated the sustainable production of plant nanoparticles decorated with nanobodies for diagnostic and therapeutic purposes.

### 5.7. Plant Viruses and VNPs Used for Cancer Immunotherapy

VLPs are evolutionarily conserved structures that mimic pathogens and can act as pathogen-associated molecular patterns (PAMPs). They are non-infectious and can induce potent cellular and humoral immune response by triggering multiple signaling pathways [191]. They are an ideal platform for vaccine development and immunotherapy. Plant viruses and their VLPs can be immunogenic for mammals. The cell-surface Toll-like receptors (TLR2 and TLR4) bind mainly viral capsid proteins, while intracellular TLRs (TLR3, TLR7, TLR8, and TLR9) detect nucleic acids and trigger the innate immune response [192]. Plant viruses and their capsid proteins, as eVLPs, can also be used as cancer vaccines when administered intratumorally [193].

A functioning immune system is crucial for immunosurveillance and protection against malignant cells within the host organism, in addition to its obvious importance in fending off intruders from outside the body. Lymphoma, Kaposi’s sarcoma, and cervical carcinoma are only some of the cancers that are more likely to occur if the human immune system is compromised [194]. Tumors can evade the immune system in several ways, including by changing their antigenic profile, suppressing their expression of major histocompatibility complex (MHC) molecules, releasing antigenic peptides into the surrounding environment, or secreting immunosuppressive chemicals [195]. Physiological immune regulatory pathways are used in some of the tumor immune evasion strategies. To suppress a T-cell-mediated immune response, it is sometimes necessary to target T-cells expressing cytotoxic T-lymphocyte-associated protein 4 (CTLA-4) or programmed cell death protein-1 (PD-1). Tumor cells can also trigger apoptosis in T-cells which exhibit the Fas receptor by expressing the Fas ligand (FasL, CD95) [196].

In the case of non-Hodgkin’s B-cell lymphomas (NHL), a novel and effective drug delivery method was discovered using PVX’s binding affinity towards malignant B cells. In a mouse model of NHL, the progression of the disease was slowed when monomethyl auristatin, loaded on PVX, was administered into regions that contained malignant B cells [197]. Using a biotin/streptavidin linker, Jobsri et al. (2015) reported that PVX attached to an idiotypic (Id) tumor-associated antigen (TAA) recombinant protein resulted in a 7-fold stronger anti-Id IgG response than Id alone in a mouse B-cell lymphoma model. TLR7 was shown to be necessary for viral RNA recognition. The cytokine profile of these animals showed that IFN- and IL-12 were induced [198]. Trastuzumab (Herceptin) is a human epidermal growth factor receptor two (HER2) binding site-targeting monoclonal antibody that inhibits the development of cancer cells. Immunogenic trastuzumab-binding peptides (TBP) can stimulate the body to make antibodies that block the development of HER2-positive cancer cells. Thus, TMV particles exhibiting TBP have been engineered to stimulate such immunogenicity [199]. Trastuzumab has been shown to successfully trigger apoptosis in breast cancer cell lines once loaded onto PVX nanofilaments [200,201]. 

A protein called vimentin is present on the surface of most cells, and it has been suggested that CPMV nanoparticles might adhere to it. Due to its upregulation during tumor growth, vimentin is a promising therapeutic target for treating cancers. Stainmetz et al. showed that surface vimentin expression linked with CPMV absorption substantiated CPMV’s efficacy in detecting invasive cancer cells [202]. In mouse models of lung melanoma, ovarian cancer, colon cancer, and breast cancer, CPMV VLP nanoparticles were found to block tumor development. It was shown that CPMV reprograms the tumor microenvironment by attracting natural killer cells and neutrophils and allowing M2 to M1 antitumor macrophages to switch to their more effective M1 state [203]. Cell lysis occurs because of this innate immune cell population’s fight against the tumor. Mao et al. (2021) have recently established which Toll-like receptors (TLRs) were accountable for such features [193]. Effective lymphatic delivery of the human epidermal growth factor receptor 2 (HER2) epitope was accomplished after conjugation to the icosahedral CPMV, resulting in increased absorption and activation of APCs and a consequently increased anti-HER2 immune response. In animal research, the CPMV HER2 candidate vaccination prolonged longevity by decreasing tumor growth and metastasis. Notably, in mouse models, CPMV-HER2 elicited a largely Th1 immune response, while Sesbania Mosaic Virus-HER2 and CCMV-HER2 elicited a predominantly Th2 response, demonstrating that the type of the epitope carrier itself plays a crucial role in determining the Th1/Th2 bias [204]. The variation in epitope presentation between the VNPs’ surface and the capsid may be too accountable for this phenomenon [205]. The immunological response to TACAs may be improved if plant viruses were utilized to deliver these molecules. The Tn antigen (GalNAc—O-Ser/Thr)-targeting CPMV-TACA conjugates were shown to generate higher IgG titers in mouse models, suggesting stronger T-cell mediated immunity and antibody isotype switching. In studies where mouse sera were added to breast cancer cell lines, IgG binding to the Tn antigens was detected [206]. Wang and Steinmetz (2019) delved deep and discovered that a protein called CD47, which is extensively expressed on tumor cells, inhibits the activity of T cells and phagocytic cells. To stimulate an antitumor immune response, the researchers utilized a combined treatment using CD47-blocking antibodies and CPMV nanoparticles. The same research team also utilized combination treatment with modest concentrations of cyclophosphamide (CPA) and CPMV nanoparticles to effectively shrink mice tumors in vivo [207].

### 5.8. Plant Viruses VLPs-Based Display Platform for Immunogenic Peptides

Plant viruses displaying heterologous epitopes have been used in numerous animal and human clinical studies with positive results. Each of the test animals was successfully protected from a challenge with the virulent mink enteritis virus thanks to CPMV particles expressing a 17-mer neutralizing epitope from the VP2 capsid protein [57]. Antibody responses were enhanced when the peptide was presented on the interface of both the L and S coat protein subunits in a modified construct, as opposed to only the L subunit. Hemocyanin-conjugated keyhole limpet predominant IgG-2a levels pointed to early stimulation of T-helper type 1 cells (TH1) [208]. The proliferation and interferon-production of murine cells treated in vitro with virus particles corroborated these findings. The immune response in the nasal mucosa after intranasal vaccination was higher than the reaction in the blood serum. These results demonstrate that presenting peptides on viral particles can change the immune response’s bias toward a TH1 response (activation of macrophages and cytotoxic T cells) and that recombinant viruses can provide protection against systemic and mucosal infections. Predominant IgG-2a levels pointed to early stimulation of T-helper type 1 cells (TH1) [209,210]. 

Bacterial pathogen epitopes, such as those found in *Pseudomonas aeruginosa* and *Staphylococcus aureus*, have also been presented using CPMV particles. Subcutaneous immunization of mice and rats with CPMV particles showing the D2 peptide from the *S. aureus* fibronectin-binding protein elicited significant titers of fibronectin-binding protein-specific antibodies. Serum from immunized mice decreased the recombinant fibronectin-binding protein’s ability to bind to immobilized fibronectin, while rat serum was able to prevent *S. aureus* from adhering to fibronectin [211,212]. PVX was also used for displaying *S. aureus* protein A fragments on its surface. The protein A coated PVX was easily functionalized with IgG and used in biosensing of plant viruses [213].

Another example is the use of genetically engineered VLPs of Papaya mosaic virus as a seasonal flu trivalent vaccine [214,215].

These results demonstrate the potential efficacy of recombinant plant virus vaccines in preventing invasive endocarditis, septicemia, peritonitis, and bovine mastitis caused by *S. aureus*. C57BL/6 mice immunized with CPMV particles presenting a linear B-cell epitope from *P. aeruginosa* outer membrane protein F generated peptide-specific antibodies that bound complement receptors and enhanced phagocytosis of *P. aeruginosa* by human neutrophils in vitro [212,216].

Tobacco mosaic VLPs have been used as epitope display systems for the first time in the production of a polio vaccine [217]. Since then, they have also been utilized in the production of various vaccines, such as malaria, human papillomavirus, rabies, hepatitis B, influenza, HIV, norovirus, and foot and mouth disease virus [211,218,219,220,221].

## 6. Methods of Application of Vaccines Produced in Plants

Different vaccine delivery methods include injection, oral, or intranasal administration. In general, the delivery of human vaccines is by injection. In veterinary medicine, when immunizing large groups of animals, many factors determine the most effective immunization method. The vaccines for the big farm animals such as cattle, sheep, goats, and pigs are delivered parenterally. Those for use in poultry are delivered by injection or orally. Oral immunization is the most preferred vaccination method for fish and other aquaculture animals. Generally, oral vaccination provides a time-saving and non-stressful application for both animals and humans [222]. However, the oral administration of vaccines has some disadvantages. Delivering a specific dose of the vaccinogen is difficult, especially when it is a non-purified recombinant immunogenic protein within a living plant [223,224]. Additionally, the vaccinogen must pass through the stomach with its low pH and digestive enzymes and retain its immunogenicity. VLPs or other highly organized particles have been shown to be more resistant to the stomach’s digestive enzymes. They are more likely to retain their immunogenicity, inducing a strong immune response in small animals [225,226]. VLPs produced in plants may represent a cost-effective approach to induce mucosal immunity by oral delivery [227]. Although the commercial implementation of this strategy in the developed world faces several regulatory challenges, this strategy may offer an attractive option for veterinary vaccine delivery in both the developed and developing world [213]. Multiple reports have shown that plant expression systems offer very good solutions for the production of vaccines for veterinary use [17,228,229,230,231,232].

### Can Plant-Based Oral Vaccines Help the One Health Approach?

The concept of One Health aims to achieve optimal health outcomes by recognizing the interconnection between people, animals, plants, and their shared environment (CDC). The idea of producing oral vaccines in edible plants carries the potential to change the pharmaceutical industry by reducing production costs by eliminating expensive downstream purifications and cold storage. However, despite the enormous research conducted to invent edible vaccines, no licensed commercial product has been approved for human or veterinary use [233]. The possible cross-contamination of nearby growing food crops with recombinant genes stifled the efforts to produce vaccines in edible crops [18]. Efforts then turned to microalgae and certain plant species, which are generally recognized as safe (GRAS) by the FDA, resurrecting the hope that oral vaccines can be produced in photosynthetic organisms without processing [234,235,236]. Edible vaccines can stimulate mucosal immunity and might be the solution for reducing zoonotic diseases distributed by wild animals and an efficient method for vaccinating large groups of animals, especially aquatic animals [228,237,238,239].

Many immunogenic proteins (viral proteins and bacterial toxins) have been expressed in plants and algae. Animal immunization studies have demonstrated the efficiency of edible vaccines in stimulating the immune system. Table 2 summarizes the achievements in producing edible vaccines in plants and algae for veterinary application.

## 7. Products, Market Size with Some Examples

From 2018 to 2026, the worldwide plant-based biologics market is expected to rise at a compound annual growth rate (CAGR) of 6.1%, reaching USD 162.4 million by 2026. The European plant-based biologics market is forecasted to grow at a CAGR of 8.1% for the same period as mentioned above [252]. The plant-based biologics products are the result of state-of-the-art studies and possess numerous advantages in terms of product safety, production scalability, easy storage, fast research and development process, and provide the opportunity to supply low-cost biologics to the low income countries. The growing demand for biological products cannot be fully met by current production platforms such as yeast, *E. coli*, insect cells, Chinese hamster ovary (CHO) cells, and embryonated hen’s eggs (EHE), due to limitations in scalability and in some cases higher cost. The plant expression systems imposed themselves as a superior alternative to conventional production systems due to increased yield of recombinant proteins, good stability, improved glycosylation patterns, and downstream processing [253,254,255,256,257,258].

The rapid development of novel biologics (vaccines, diagnostic reagents, and therapeutics) are among the main objectives of companies such as Protalix Biotherapeutics (Carmiel, Israel); Medicago Inc. (Québec, Canada); iBio/Caliber Therapeutics (Bryan, TX, USA); Kentucky BioProcessing Inc. (Owensboro, KY, USA; Fraunhofer USA (Plymouth, MI, USA); Pfizer (New York, NY, USA); Baiya Phytopharm (Khwaeng Prawet, Prawet, Bangkok, Thailand); Leaf Expression Systems (Norwich Research Park, Norwich, UK), Cape Biologix Technologies (Ndabeni, Cape Town, South Africa); Zea Biosciences (Walpole, MA, USA); Planet Biotechnology Inc. (Hayward, CA, USA); Mapp Biopharmaceutical Inc. (San Diego, CA, USA); and others. The breakthrough in plant-made vaccines was made by Dow AgroSciences, receiving the first USDA approval for a vaccine against NDV, based on the HN protein produced in an *N. benthamiana* cell culture [24]. Protalix Biotherapeutics received the first FDA approval for commercial use in humans of their plant-made recombinant human enzyme β-glucocerebrosidase for Gaucher disease. Pfizer received the license for this product and marketed it as Elelyso^®^ [259]. Mapp Biopharmaceutical Inc. was granted fast-track designation by the FDA for their plant-made cocktails of three monoclonal antibodies (ZMapp) against Ebola virus disease [260,261]. We have even witnessed the first licensed plant-derived vaccines Covifenz against influenza and SARS-CoV-2 developed by Medicago [26].

The major players in PMF have a number of biopharmaceutical products in clinical trials or products that are awaiting approval from regulatory authorities. The American biotech company iBio Inc. has four plant-based biologics in preclinical trials. IBIO-100 targets fibrosis, IBIO-200 is a virus-like particle (VLP) vaccine against SARS-CoV-2, IBIO-201 is a vaccine targeting COVID-19 using the patented LicKM platform, and IBIO-400 is a vaccine targeting classical swine fever (CSF) disease [262]. Furthermore, KBP [263] and Baiya Phytopharm [264] announced that they have produced subunit vaccines against SARS-CoV-2 in plants [265].

Proteins with antibacterial properties can be used as medications and/or as antibacterial food additives. Using GRAS (Generally Recognized As Safe) regulatory procedures, Nomad Bioscience GmbH (Nomad) has been allowed by the United States Food and Drug Administration (FDA) to use *Escherichia*-derived (colicins) and Salmonella-derived (salmocins) antibacterial proteins in treatments of fresh or processed fruits and vegetables (colicins), or meat, poultry, fish, and whole eggs (salmocins). These bacteriocins, produced in plant expression systems, have been proven highly effective, even when used in low concentrations, for controlling pathogenic bacteria in food products and avoiding food poisoning. In addition, their production is also financially justifiable [266,267].

Clinical trials of innovative biological treatments such as nanobodies, recombinant vaccines, fusion proteins, antisense RNAi therapies, and gene and cell therapy are underway. Hence, such product developments are expected to positively impact the growth of the global plant-based market.

## 8. Challenges in Developing Biopharmaceuticals in Plants

Among the main challenges of using plants and different plant viruses as expression platforms for human pharmaceuticals are the plant glycosylation pathways resulting in distinct plant-specific glycans from those in humans or for that matter in most mammalian glycoproteins. The concern is that epitopes carrying non-human glycoforms (β1,2-xylose and α1,3-fucose) would be regarded as foreign by the immune system and hence deemed to be more immunogenic in humans resulting in mild or hypersensitive allergic response [268]. However, it should be noted that these concerns were alleviated to some extent by recent safety data on Medicago Inc. influenza and SARS-CoV-2 vaccines clinical trials phase 3. Volunteers, some of whom had existing plant allergies, were followed for six months and none developed allergic or hypersensitivity reactions.

Potentially, the impact of non-human glycoforms may be more relevant with plant monoclonals as they are usually administered intravenously in mg quantities compared to ug in vaccines or other pharmaceuticals. Still, it is not known if repeated immunizations, as is the case with the annual flu vaccine, or repeat potential boosters, for instance with plant-derived Medicago SARS-CoV-2 vaccine, may have undesirable immune responses due to plant glycans. On the other hand, the potential immunogenicity of plant glycans in some cases could be beneficial, for instance with cancer vaccines/immunotherapeutics enhancing their effect [269].

On the opposite spectre of unwelcome impact due to the expanded immunogenicity of plant glycoproteins is their potential rapid clearance by the immune system which would diminish their efficacy as pharmaceuticals. Conversely, some native, complex mammalian glycoproteins may have a detrimental impact on the plant cellular glycosylation mechanisms leading to endoplasmic reticulum-related stress response demonstrated by leaf necrosis that ultimately results in low yield of recombinant proteins. To alleviate the majority of the above mentioned drawbacks, different glycoengineering strategies have been recently developed to undo undesirable immunogenic N-glycan modifications in plants such as (i) “humanization” of plant *N*-glycans by production of knock-out lines removing genes responsible for plant-specific glycosylation by using the CRISPR/Cas9 system, (ii) introduction of humanized N-glycosylation pathways in plants, and (iii) co-expression of mammalian native chaperons and folding enzymes compatible with the plant endogenous chaperone machinery. This is an incomplete set of glycoengineering tools to modify plant glycosylation pathways in order to improve tomorrow’s plant pharmaceuticals.

## 9. Conclusions

Since their discovery, plant viruses have been transformed into a major molecular tool used for recombinant protein expressions, vaccine manufacturing, and as nonoagents for drug delivery. Whole virions, naturally occurring empty capsids of plant viruses, nanoparticles generated by reassembly of coat proteins, and VLPs displaying heterologous peptides or even whole proteins on their surface or inside their cavity are all examples of plant-made nanoparticles used for vaccine and biologics production, and in nanoscale drug delivery applications. Unlike the mainstream expression platforms, the production of plant-based biologics has its advantages: absence of major safety concerns, no need for expensive equipment and continuous temperature control, easy scalability, and affordability. Several promising plant-derived biologics are being tested in clinical trials, which should lead to cutting-edge therapies in the rapidly growing field of VNP- and VLP-based drug delivery applications.

## Figures and Tables

**Table 1:** List of some viruses used for the design of viral vectors.

Virus Classification/Genome Organization	Virus	Vector	Achieved High Yield	Recombinant Proteins	Reference
Tobamovirus + ssRNA	tobacco mosaic virus (TMV)	magnICON	5 mg/g FWT	GFP	[88]
		TRBO	5.5 mg/g FWT	GFP	[89]
Potexvirus + ssRNA	foxtail mosaic virus (FoMV)	FECT/40 vector	1.7 mg/g FWT	GFP	[90]
	potato virus X (PVX)	pEff	1 mg/g FWT	GFP	[84]
Geminivirusss circular DNA	tobacco yellow dwarf mastrevirus (TYDV)	INPACT	0.1 mg/g FWT	human Vitronectin	[91]
	bean yellow dwarf virus (BeYDV)	pBYRp19	0.5 mg/g FWT	mAb 6D8 against Ebola virus	[92]
		pRIC	0.55 mg/g FWT	HPV CP L1	[93]
Tobravirus + ssRNA	tobacco rattle virus (TRV)	TRV- based vector	0.01 mg/g of fresh weight root tissue	GNA lectin protein	[94]
Comovirus + ss RNA	cowpea mosaic virus (CPMV)	delRNA-2	-	GFP	[95]
		pEAQ-*HT*	1.5 mg/g	GFP	[96]

**Table 2:** List of known to be edible vaccines against animal pathogens.

Disease/Infectious Agents	Antigen	Species	Yield	Immunogenicity	Reference
Norwalk virus	Capsid protein (NVCP)	Tomato	up to 8% of TSP	Freeze-dried tomato (40 µg VLPs) induced NV-specific serum IgG and mucosal IgA in ≥80% of mice.	[240]
		Potato	-	19/20 human volunteers developed an immune response after oral immunization with VLPs.	[241]
Rabies virus	G and N proteins fused to AlMV CP	Tobacco and spinach	0.4 ± 0.07 mg/g of fresh leaf tissue	Immunized mice were protected against challenge infection. Human volunteers previously non-immunized demonstrated significant antibody responses after fed.	[242]
Newcastle Disease Virus (NDV)	Hemagglutinin-neuraminidase protein (HN)	Tobacco	0.069% of TSP	Specific immune response after oral administration of chicken was induced.	[243]
	Fusion (F)	Maize	0.9–3% TSP		[244]
	Fusion (F) and hemagglutinin-neuraminidase (HN) proteins	Maize	0.5–0.8% of total seed protein		[245]
		Canola	up to 0.18% and 0.11% TSP in transgenic seeds and leaves		[246]
		Potato	0.3–0.6 mg/g of total leaf protein		[247]
*S. aureus* and Cholera	D2 fibronectin-binding domain with cholera toxin B subunit	*C. reinhardtii*	Up to 0.7% TSP	Mice fed with whole algae showed mucosal IgA and systemic IgG responses to CTB and D2. A total of 80% survived after lethal challenge.	[248]
Bovine rotavirus (BVR)	eBRV4 fused to βGUS	Alfalfa	0.4–0.9 mg (g TPS)^−1^	An effective anti-rotavirus antibody response was induced in mice after oral administration.	[249]
Coronavirus	S protein (S1)	Tomato	-	Orally immunized mice showed significantly increased levels of SARS-CoV-specific IgA.	[250]
Hog pest virus/*F. hepatica*	Glycoprotein E2/cysteine proteases	Lettuce	0.16 mg/g dry mass	Oral immunization of mice induced specific antibodies.	[251]
